# Solid-phase enrichment uncovers a hidden *Salmonella* transmission chain in a recurrent pediatric household cluster: a case report

**DOI:** 10.3389/fpubh.2026.1820049

**Published:** 2026-05-19

**Authors:** Feng Tang, Hua Liu, Lei Xi, Changzhen Li, Xiaomei Wang, Baoxiang Wang

**Affiliations:** 1 Department of Laboratory Medicine, Wuhan Children's Hospital (Wuhan Maternal and Child Healthcare Hospital), Tongji Medical College, Huazhong University of Science & Technology, Wuhan, Hubei, China; 2 Department of Gastroenterology, Wuhan Children's Hospital (Wuhan Maternal and Child Healthcare Hospital), Tongji Medical College, Huazhong University of Science & Technology, Wuhan, Hubei, China

**Keywords:** antibiotic-induced dysbiosis, household transmission, metagenomic sequencing, non-typhoidal *Salmonella*, pediatric recurrent infection, *Salmonella* Enteritidis, solid-phase enrichment, whole-genome sequencing

## Abstract

**Objectives:**

To describe a household cluster of recurrent pediatric non-typhoidal *Salmonella* (NTS) infection and compare the yield of conventional culture, solid-phase enrichment, and shotgun metagenomic sequencing across symptomatic children and household contacts.

**Methods:**

Longitudinal fecal specimens from a 4-year-old boy (Mo) with three discrete NTS episodes in 2 months, his monozygotic twin (TB), and three adult co-residents were processed by conventional culture; specimens from Episode 2 onwards and all contact specimens additionally received solid-phase enrichment, and a subset shotgun metagenomics. Isolates were characterized by VITEK 2, XbaI-PFGE, and whole-genome sequencing.

**Results:**

None of Mo's episodes met sepsis criteria (peak WBC 12.52 × 10?/L, CRP 5.46 mg/L, PCT 1.14 ng/mL); TB had one self-limited episode, both parents had brief symptomatic periods, and the grandmother was asymptomatic. Conventional culture was positive only at Mo's first episode, whereas solid-phase enrichment recovered Salmonella from three culture-negative pediatric acute-phase specimens (Mo 4.12, TB 4.16, Mo 5.1). Adult contacts were negative by both culture-based methods, but metagenomic sequencing detected *Salmonella* reads in all three. Mo_0412 and TB_0416 were *S. enterica* serovar Enteritidis ST11, with identical cgMLST, 99.9966% ANI, and 97% PFGE similarity, indicating a clonal household source. Mo received antibiotics across four classes during his recurrences, vs. two sequential agents in TB.

**Conclusion:**

Conventional culture, solid-phase enrichment, and metagenomic sequencing functioned as complementary modalities, each recovering *Salmonella* the others missed, supporting a tiered diagnostic strategy for household NTS investigation. Cumulative antibiotic exposure may have contributed to Mo's differential susceptibility, a hypothesis warranting prospective study.

## Introduction

Non-typhoidal *Salmonella* (NTS) infections are a leading cause of pediatric diarrheal illness globally, accounting for an estimated 93.8 million episodes annually (1). Global burden-of-disease analyses rank non-typhoidal *Salmonella* among the principal etiologies of diarrhea-related mortality across all age groups (2). Recurrent episodes in the same child present significant management challenges and frequently signal unresolved household exposure rather than intrinsic host susceptibility alone (3, 4). Family members who acquire NTS asymptomatically can serve as occult reservoirs perpetuating intrafamilial transmission cycles, yet standard microbiological investigation often fails to identify these individuals (5).

The sensitivity of direct plating from fecal specimens for *Salmonella* detection in low-burden settings has been estimated at 62%−70%, rendering conventional culture an unreliable tool for carrier surveillance in household contacts (6). Whole-sample solid-phase enrichment methods—which concentrate target organisms from the entire fecal matrix prior to selective plating on chromogenic media—offer substantially improved yields from low-burden specimens and represent an important methodological advance for household outbreak investigation (7, 8).

We describe a case of recurrent pediatric *Salmonella* infection in which conventional microbiological investigation failed to identify a household transmission source, in which solid-phase enrichment recovered *Salmonella* from three pediatric acute-phase specimens (Mo 4.12, TB 4.16, and Mo 5.1) that had been negative by conventional culture but did not recover it from household contacts, and in which exploratory metagenomic sequencing subsequently detected low-level *Salmonella*-assigned reads in fecal specimens from all household members. We discuss the complementary strengths and limitations of culture-based and sequence-based detection in this setting.

## Methods

### Study population and ethics

The family comprised 2-year-old monozygotic twin boys (Mo and TB), their parents (F and M), and maternal grandmother (GM), all co-residing in the same household in Wuhan, China. The study protocol was approved by the Wuhan Children's Hospital Ethics Review Committee (2024R05-E2); written informed consent was obtained from the legal guardians of all participants prior to sample collection.

### Conventional stool culture

Fecal specimens from all five household members were inoculated onto blood agar, MacConkey agar, and *Salmonella*-*Shigella* agar and incubated at 37 °C for 18–24 h. Suspicious colonies were subjected to species-level confirmation by matrix-assisted laser desorption/ionization time-of-flight mass spectrometry using the MALDI Biotyper system (Microflex LT/SH; Bruker Daltonics) and confirmatory biochemical identification cards.

### Solid-phase enrichment assay

Apart from the index Episode-1 specimen (Mo 3.16), which was diagnostic by routine culture alone, all subsequent fecal specimens were processed in parallel with conventional culture by an optimized whole-sample solid-phase enrichment method previously developed and validated by our group (7, 8). Briefly, homogenized fecal material was applied to a functionalized solid-phase capture matrix, incubated under standardized temperature and agitation conditions, and eluate was selectively plated on *Salmonella* chromogenic medium. By concentrating target organisms from the entire specimen volume rather than a direct surface aliquot, this approach enables recovery from samples below the limit of detection of conventional direct plating.

### Antimicrobial susceptibility testing

Sample inoculation, culturing, and subsequent testing were performed in accordance with the “Basic Technical Standard for Clinical Microbiology Laboratory” (People's Republic of China Health Industry Standard WS/T 805–2022). Antimicrobial susceptibility of all *Salmonella* isolates recovered from the cluster was determined using the VITEK 2 Compact system (bioMérieux, Marcy-l'Étoile, France) following the manufacturer's instructions, complemented by the Kirby–Bauer disk diffusion method for agents not covered by the automated panel. Bacterial suspensions were prepared in 0.45% saline and adjusted to a 0.5 McFarland standard (DensiCHEK Plus, bioMérieux) prior to inoculation. The tested panel comprised 12 antibiotics: ampicillin (AMP), levofloxacin (LEV), ciprofloxacin (CIP), trimethoprim-sulfamethoxazole (SXT), ceftriaxone (CRO), ceftazidime (CAZ), amikacin (AMK), cefepime (FEP), imipenem (IPM), ampicillin-sulbactam (SAM), piperacillin-tazobactam (PIZ), and ertapenem (ETP). Minimum inhibitory concentrations (MICs) and inhibition zone diameters were interpreted as susceptible (S), intermediate (I), or resistant (R) according to the Clinical and Laboratory Standards Institute (CLSI) breakpoint standards. *Escherichia coli* ATCC 25922 served as the quality-control strain.

### Pulsed-field gel electrophoresis

Representative isolates Mo_0412 (from Mo) and TB_0416 (from TB) were selected for molecular subtyping. Agarose-embedded genomic DNA was digested with XbaI (Takara) for 4 h at 37 °C and separated on a Bio-Rad CHEF-DR III apparatus for 18–19 h. *Salmonella enterica* serovar Braenderup H9812 served as the molecular size standard. Band patterns were analyzed with BioNumerics v7.6 (Applied Maths) using the dice coefficient, and the ≥85% band-pattern similarity cutoff was applied to define a PFGE cluster (9).

### Whole-genome sequencing and bioinformatic analysis

Mo_0412 and TB_0416 were further subjected to paired-end Illumina whole-genome sequencing. Genomic DNA was extracted from overnight cultures and used for library preparation; libraries were sequenced on an Illumina platform in paired-end mode. Raw-read quality assessment, adapter and quality trimming, *de novo* assembly, and assembly-quality evaluation were all performed in CLC Genomics Workbench version 25.0.3 (QIAGEN Digital Insights) using default parameters. Genomic characterization of the assembled genomes was then performed using the following standard bioinformatic tools. *In silico* serotyping (serovar, antigenic formula, serogroup) and core-genome multilocus sequence typing (cgMLST) were performed in a single SISTR run. Seven-gene multilocus sequence typing (MLST) was assigned with the mlst tool (T. Seemann) against the PubMLST *Salmonella* scheme. Pairwise whole-genome average nucleotide identity (ANI) was calculated with FastANI. Acquired antimicrobial resistance genes, plasmid replicons, and virulence factors were identified with ABRicate by screening the assembled genomes against the ResFinder, PlasmidFinder, and Virulence Factor Database (VFDB) databases, respectively; VFDB hits were subsequently grouped by VFDB functional category.

### Exploratory metagenomic profiling

**Figure 1:**
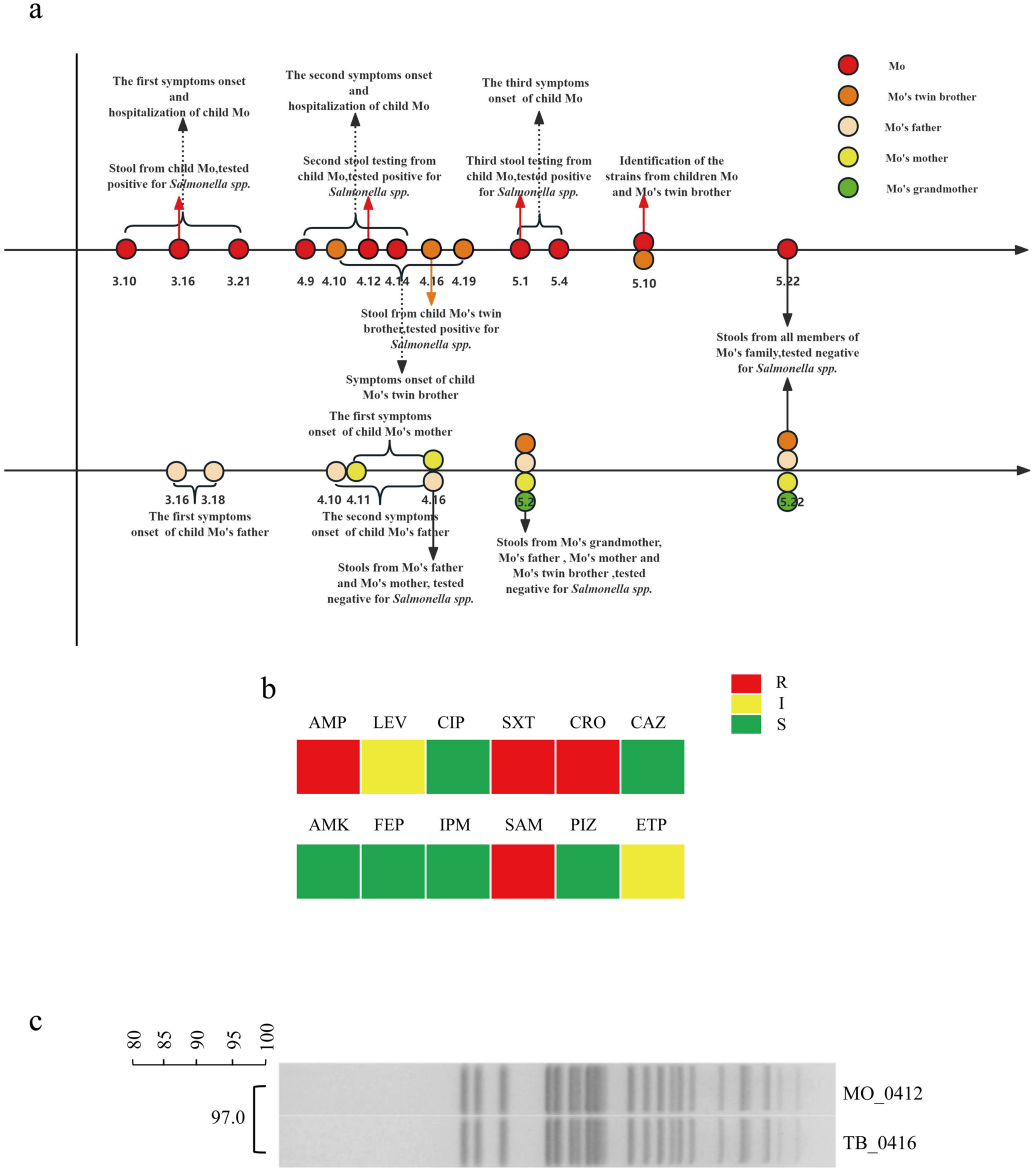
Household transmission timeline, antimicrobial susceptibility profiles, and PFGE-based banding-pattern analysis of *Salmonella* isolates recovered from the household cluster. **(a)** Clinical and microbiological timeline of the outbreak. The upper axis shows events for the index child (Mo, red) and his monozygotic twin brother (TB, orange); the lower axis shows events for the father (F, beige), mother (M, yellow), and maternal grandmother (GM, green). Each colored circle represents a stool-sampling event on the indicated date (month.day). For Mo and TB, positivity for *Salmonella* was established by conventional stool culture (Mo 3.16 only) or by solid-phase enrichment after conventional culture returned negative (Mo 4.12, TB 4.16, and Mo 5.1); the corresponding sampling events are annotated with vertical arrows colored to match each subject (red for Mo, orange for TB) labeled “tested positive for *Salmonella* spp.” During the outbreak window, F experienced two discrete episodes of self-limited gastrointestinal symptoms (3.16–3.18 and 4.10–4.16), M experienced a single episode of abdominal discomfort and bowel urge (4.11–4.16), and GM remained asymptomatic throughout, all as annotated on the lower axis. For F, M, and GM, conventional stool culture was negative at every sampling event throughout the outbreak (black downward arrows annotated “tested negative for *Salmonella* spp.”), and parallel testing of the same specimens by the optimized whole-sample solid-phase enrichment assay was also negative at all timepoints. Vertical arrows linked to text annotations also indicate symptom onsets, hospital admissions, and strain-identification milestones. Horizontal brackets above the Mo and TB rows delineate the antibiotic courses administered during the observation period: three courses for Mo, corresponding to his three recurrences and cumulatively spanning four distinct antibiotic classes (penicillin, clindamycin, ceftazidime, and metronidazole), and one course for TB, comprising sequential clindamycin and metronidazole during his single episode (two antibiotic classes). **(b)** Antimicrobial susceptibility profile determined on two representative *Salmonella* isolates from the cluster (Mo_0412 from Mo's second episode and TB_0416 from TB's episode); the remaining isolates (Mo_0316 and Mo_0501) were not subjected to full panel testing. Red, resistant **(R)**; yellow, intermediate **(I)**; green, susceptible **(S)**; Antibiotics tested: AMP, ampicillin; LEV, levofloxacin; CIP, ciprofloxacin; SXT, trimethoprim-sulfamethoxazole; CRO, ceftriaxone; CAZ, ceftazidime; AMK, amikacin; FEP, cefepime; IPM, imipenem; SAM, ampicillin-sulbactam; PIZ, piperacillin-tazobactam; ETP, ertapenem. The fully concordant resistance phenotype between the two tested isolates is consistent with a common-source cluster. **(c)** Pulsed-field gel electrophoresis (XbaI-digested genomic DNA) of representative isolates Mo_0412 (from Mo) and TB_0416 (from TB). BioNumerics v7.6 dice-coefficient analysis indicated 97% band-pattern similarity, exceeding the ≥85% threshold conventionally used to define a PFGE cluster. Whole-genome sequencing confirmation of clonal identity between the two isolates is provided in Figure 3 and Table 1.

For longitudinal gut microbiota profiling, 23 fecal samples were collected from all five household members across acute-phase (AP) and reservoir-phase (RP) timepoints (see Figure 1c for the sample list). Shotgun metagenomic sequencing and bioinformatic analysis were performed by BGI (Shenzhen, China) following their standardized workflow. In brief, total DNA was fragmented by ultrasonication (Covaris, Woburn, MA, USA), size-selected to 200–400 bp, end-repaired, A-tailed, adapter-ligated, PCR-amplified, and circularized to yield single-stranded DNA nanoball (DNB) libraries. Libraries were sequenced on a DNBSEQ / MGISEQ 2000 platform in paired-end 150 bp (PE150) mode. Raw reads were quality-filtered and adapter-trimmed using SOAPnuke, and human host reads were removed by mapping against the human reference genome with Bowtie 2 to generate clean data. Clean reads were assembled per sample with MEGAHIT (contigs < 300 bp discarded); genes were predicted on assembled contigs with MetaGeneMark and dereplicated with CD-HIT to build a non-redundant gene catalog. Gene abundance was estimated with Salmon. Taxonomic profiling was performed with Kraken 2 against a custom database combining NCBI nt and the Unified Human Gastrointestinal Genome (UHGG) catalog, and species-level relative abundances were re-estimated with Bracken 2. Functional annotation was performed by aligning the non-redundant gene set against eggNOG, KEGG, CARD, BacMet, COG, CAZy, and Swiss-Prot using DIAMOND. Linear discriminant analysis effect size (LEfSe) was used to identify taxa nominally differentiating AP from RP samples, with default significance thresholds (Kruskal–Wallis α = 0.05; pairwise Wilcoxon α = 0.05; LDA score > 2.0).

## Results

### Clinical presentation and course

**Figure 2:**
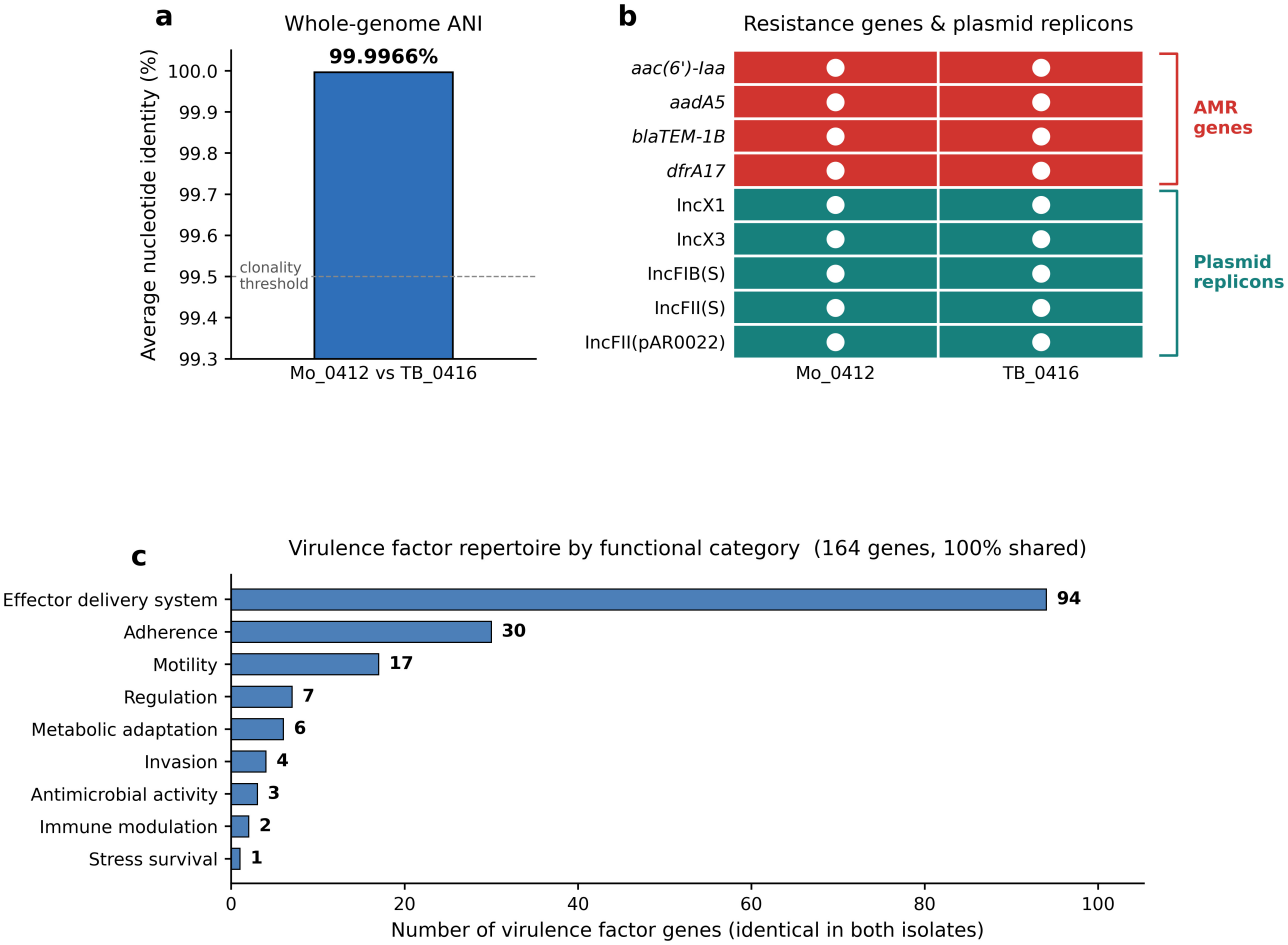
Whole-genome sequencing evidence for clonal identity between *Salmonella enterica* serovar Enteritidis isolates Mo_0412 and TB_0416. Both isolates were subjected to paired-end Illumina short-read whole-genome sequencing and characterized using a standardized bioinformatic pipeline. Comprehensive strain-typing results (serovar, antigenic formula, MLST sequence type with allelic profile, cgMLST sequence type) together with sequencing quality metrics are provided in Table 1. **(a)** Pairwise average nucleotide identity (ANI) between Mo_0412 and TB_0416, calculated with FastANI, was 99.9966%, well above thresholds typically used to infer clonality. The dashed horizontal line marks a 99.5% reference level. **(b)** Acquired antimicrobial resistance genes (red) and plasmid replicons (teal) detected in each isolate, identified by ABRicate (T. Seemann) by screening the assembled genomes against the ResFinder and PlasmidFinder databases, respectively. White dots denote presence. Both isolates carried an identical set of four resistance genes (*aac(6*′*)-Iaa, aadA5*, *bla*_TEM − 1B_, *dfrA17*) and five plasmid replicons [IncX1, IncX3, IncFIB**(S)**, IncFII**(S)**, IncFII(pAR0022)]. **(c)** Virulence-factor gene content grouped by functional category (Virulence Factor Database, VFDB, 2022 release; queried via ABRicate). A total of 164 virulence genes were identified in each isolate, with 100% concordance between the two genomes; bars show the number of genes per category (identical in both isolates). The combined evidence—near-identical whole genomes (ANI 99.9966%), identical MLST and cgMLST profiles, identical accessory-genome content, and fully concordant virulence-factor repertoires—establishes clonal identity between the two isolates, complementing the 97% band-pattern similarity observed by PFGE (Figure 2c) and supporting a common-source intrafamilial transmission event between Mo and TB.

The index patient, Mo, a 4-year-old boy, presented in March with high-grade fever (peak 39.4 °C on 3.10), watery diarrhea that progressed to mucus-bloody stools (7–8 egg-drop-like stools/day on 3.10, escalating to ≥10 mucus-bloody stools/day by 3.13), and hematochezia; stool routine on 3.16 showed WBC 3–6/HP with RBC >40/HP and occult blood OB(+++), and stool culture confirmed non-typhoidal *Salmonella* (NTS) infection. Prior to admission to our hospital on 3.15, Mo had received empirical penicillin (3.10) and clindamycin (3.12, during a brief admission to a regional hospital in Xiaogan); on admission to our hospital, laboratory findings included peripheral WBC 7.68 × 10?/L, CRP 5.46 mg/L, and PCT 1.14 ng/mL, and he was treated with ceftazidime (3.15–3.21). He improved clinically, with CRP and PCT normalizing within 72 h (CRP < 0.78 mg/L, PCT 0.32 ng/mL, peripheral WBC 9.09 × 10?/L, and stool routine WBC 0–1/HP with OB(±) on 3.18–3.19). Mo subsequently experienced two further discrete acute NTS episodes of decreasing severity. His second episode (4.9–4.14) was afebrile and characterized by soft bloody stools approximately once daily; stool routine on 4.12 showed WBC 15–20/HP, RBC 10–15/HP, and OB(++), with peripheral WBC 12.52 × 10?/L and CRP 4.0 mg/L (PCT not measured), and was managed with metronidazole 0.12 g BID for 8 days, after which stool routine, CRP, and peripheral WBC had normalized (4.19: WBC 11.13 × 10?/L, CRP < 0.78 mg/L; stool routine negative). His third episode (5.1–5.4) was the mildest, characterized by low-grade fever (peak 37.9 °C on 5.2), five formed stools on 5.1 (one bloody), and stool routine WBC 6–8/HP, RBC 1–3/HP with OB(+); peripheral WBC, CRP, and PCT were not remeasured during this episode. Episode 3 was managed with a single day of clindamycin (5.2) together with an oral berberine-compound preparation. Conventional stool culture was performed on each specimen and was positive for the Episode-1 specimen (3.16); the Episode-2 and Episode-3 specimens were also processed in parallel by solid-phase enrichment, and both the Episode-2 isolate (Mo_0412) and the Episode-3 isolate (Mo_0501) were recovered by solid-phase enrichment after parallel conventional culture had returned negative. His monozygotic twin brother TB experienced a single episode (4.10–4.19), comprising 4–5 watery stools on 4.10, bloody stools on 4.11 and again on 4.18, and return to normal stools by 4.20; TB remained afebrile throughout. His 4.16 stool specimen was conventional-culture negative but solid-phase-enrichment positive [stool routine on 4.16 was normal with OB(+)]. TB received an 8-day course of clindamycin (commenced 4.10) followed by metronidazole 0.12 g BID commenced on 4.19 (peripheral WBC 9.7 × 10?/L on 4.19). Discrete episodes were defined operationally as complete resolution of acute symptoms with normalization of stool routine and occult-blood tests for ≥2 weeks, followed by new-onset acute gastrointestinal symptoms accompanied by a new positive stool test. Blood cultures were not obtained; no clinical or laboratory features of sepsis or invasive NTS disease were observed across the three episodes (peak PCT 1.14 ng/mL, below the ≥2 ng/mL threshold; peak peripheral WBC 12.52 × 10?/L; no hemodynamic instability, respiratory distress, or altered mental status documented). No external common exposure source (e.g., shared meal, institutional outbreak, or travel history) was identified across the 3-month observation period. The sequential clinical and microbiological events for all five household members are summarized in Figure 2a.

### Conventional culture of household contacts was negative

During the outbreak window, F experienced two discrete episodes of self-limited gastrointestinal symptoms (3.16–3.18, with 3–4 loose stools/day and a single bloody stool on 3.16; and 4.10–4.16, with 1–3 loose stools/day and a single bloody stool on 4.12). M experienced a single brief episode of abdominal discomfort and bowel urge (4.11–4.16), not accompanied by fever, frank diarrhea, or hematochezia. GM remained asymptomatic throughout the observation period. In parallel with Mo's recurrences, stool specimens from F, M, and GM were submitted for conventional stool culture. All three household contacts returned negative results for *Salmonella* on every sampling occasion (Figure 2a). Without a positive contact result, the treating team could not identify an ongoing household reservoir, and the working assumption initially remained that Mo's recurrences reflected re-infection from an unidentified external source.

### Solid-phase enrichment recovered *Salmonella* from culture-negative pediatric specimens

The parallel solid-phase enrichment workflow recovered *Salmonella* from three culture-negative pediatric specimens—Mo's 4.12, TB's 4.16, and Mo's 5.1—bringing the total number of microbiologically confirmed cluster isolates to four (Mo_0316 by conventional culture; Mo_0412, TB_0416, and Mo_0501 by solid-phase enrichment after parallel conventional culture had returned negative). The same enrichment workflow, however, did not recover *Salmonella* from any of the repeatedly sampled specimens from F, M, and GM (Figure 2a). Exploratory shotgun metagenomic sequencing of the longitudinal fecal series (Figure 1) subsequently detected low-abundance *Salmonella*-assigned reads in samples from all three household contacts that had been negative by both conventional culture and solid-phase enrichment, a descriptive signal consistent with household-wide low-burden exposure but insufficient on its own to establish viable carriage.

### Antimicrobial susceptibility profiles are fully concordant

Antimicrobial susceptibility profiles were determined on two representative *Salmonella* isolates from the cluster: Mo_0412 (from Mo's second episode) and TB_0416 (from TB's episode). Susceptibility phenotypes were identical between the two isolates (Figure 2b). The fully concordant profiles are consistent with the common-source cluster hypothesis; divergence in resistance profiles would have argued against clonal intra-familial spread.

### PFGE indicates a common-source cluster

Representative isolates Mo_0412 and TB_0416 demonstrated 97% PFGE band-pattern similarity (Figure 2c), exceeding the ≥85% threshold conventionally used to define a PFGE cluster (9). This molecular evidence supports the interpretation that Mo and TB acquired NTS from a common intra-familial source, complementing the epidemiological concordance of Mo's and TB's April admissions. To provide definitive clonal resolution, we next performed whole-genome sequencing of both isolates.

### Whole-genome sequencing confirms clonal identity

**Figure 3:**
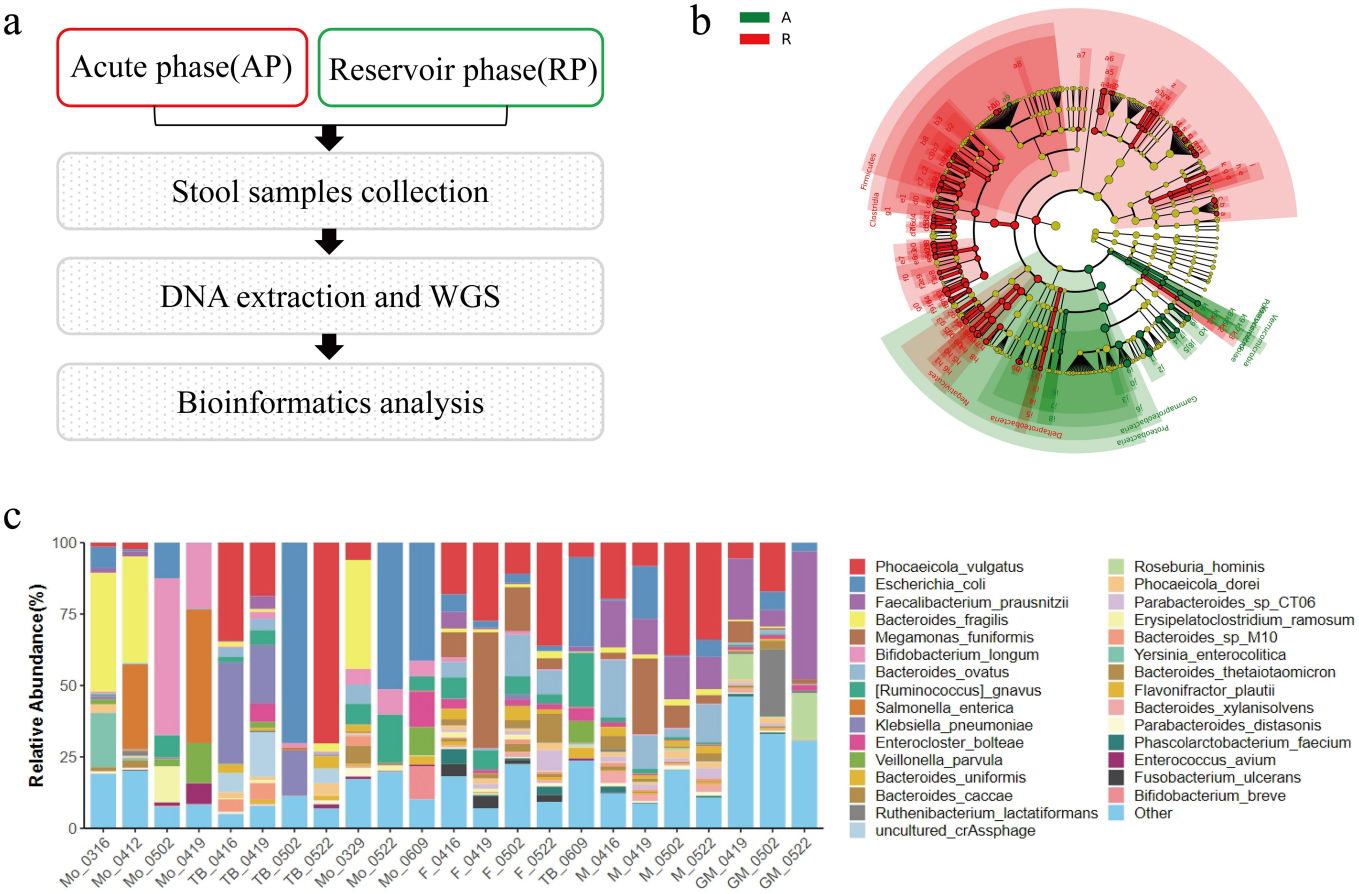
Exploratory longitudinal gut microbiota profiling of household members across acute and reservoir phases of the *Salmonella* outbreak. **(a)** Study workflow. Stool specimens from all five household members were classified as acute-phase (AP; red border in the schematic) or reservoir-phase (RP; green border) based on the clinical timeline in Figure 2a, and processed through a uniform pipeline of stool collection, DNA extraction, shotgun metagenomic sequencing, and bioinformatic analysis. **(b)** LEfSe cladogram of microbial taxa nominally discriminating acute-phase from reservoir-phase fecal samples. In the key, “A” denotes acute-phase-enriched taxa (green) and “R” denotes reservoir-phase-enriched taxa (red). Each node represents a taxon; node color indicates the phase in which the taxon is enriched, and node size reflects the linear discriminant analysis (LDA) effect magnitude. Note that the AP/RP color assignment in panel **(b)** follows the LEfSe default and is inverted relative to panel **(a)** [panel **(a)** AP red, RP green; **(b)** AP green, RP red]. **(c)** Species-level relative abundance of the gut microbiota across 23 longitudinal fecal samples from the five household members (Mo, TB, F, M, GM); samples are labeled by subject and sampling date (month.day). Notable features include marked depletion of butyrate-producing commensals (*Faecalibacterium prausnitzii, Roseburia hominis*) and relative expansion of *Escherichia coli* in Mo's acute-phase samples compared with his reservoir-phase samples and with samples from other household members, a pattern compatible with antibiotic-mediated dysbiosis. These microbiota findings are presented as hypothesis-generating and descriptive only. Given the single-household design and limited sample size, no causal or mechanistic conclusions should be drawn. Confirmation in larger prospective cohorts with appropriate controls is required.

Both Mo_0412 and TB_0416 genomes were sequenced to high quality (Q20 ≥ 99.1%; Q30 ≥ 75.2%; 2.34 Gb and 1.96 Gb of paired-end data, respectively; Table 1). *In silico* serotyping identified both isolates as *Salmonella enterica* serovar Enteritidis (antigenic formula 1,9,12:g,m:-; serogroup D1). MLST assigned both isolates to sequence type ST11, with identical seven-gene allelic profiles (*aroC*-5, *dnaN*-2, *hemD*-3, *hisD*-7, *purE*-6, *sucA*-6, *thrA*-11); cgMLST likewise yielded identical sequence types (3610863218). Pairwise average nucleotide identity between the two genomes was 99.9966% (Figure 3a), well above thresholds typically used to infer clonality. Acquired resistance-gene and plasmid-replicon profiles were identical between isolates: four resistance genes (*aac(6*′*)-Iaa, aadA5*, *bla*_TEM − 1B_, *dfrA17*) and five plasmid replicons [IncX1, IncX3, IncFIB(S), IncFII(S), IncFII(pAR0022)] were detected in both genomes (Figure 3b). Virulence-factor profiling against VFDB identified 164 genes in each isolate, with 100% gene-level concordance between the two genomes across all nine functional categories examined (Figure 3c). Together, these data establish clonal identity between Mo_0412 and TB_0416, consistent with common-source intrafamilial transmission from Mo to TB and complementing the 97% band-pattern similarity observed by PFGE (Figure 2c).

**Table 1:** Whole-genome sequencing characterization of *Salmonella enterica* serovar Enteritidis isolates Mo_0412 (from child Mo) and TB_0416 (from twin brother TB) recovered from the household cluster.

Category	Metric	Mo_0412	TB_0416
Sequencing QC	Paired-end reads	16,699,210	14,681,322
	Total bases (Gb)	2.34	1.96
	Q20 (%)	99.62	99.12
	Q30 (%)	82.98	75.26
Species/Serotyping	Species (SISTR)	*Salmonella enterica*	*Salmonella enterica*
	Serovar	Enteritidis	Enteritidis
	Antigenic formula	1,9,12:g,m:-	1,9,12:g,m:-
	Serogroup	D1	D1
Molecular typing	MLST sequence type	ST11	ST11
	MLST 7-gene profile^†^	5-2-3-7-6-6-11	5-2-3-7-6-6-11
	cgMLST sequence type	3610863218	3610863218
	ANI vs. paired isolate	99.9966%	99.9966%
Acquired resistance	AMR genes (count)	4	4
	AMR genes identified	*aac(6′)-Iaa, aadA5, blaTEM-1B, dfrA17*	*aac(6′)-Iaa, aadA5, blaTEM-1B, dfrA17*
Plasmid content	Replicons (count)	5	5
	Replicon types	IncX1, IncX3, IncFIB(S), IncFII(S), IncFII(pAR0022)	IncX1, IncX3, IncFIB(S), IncFII(S), IncFII(pAR0022)
Virulence	VFDB virulence genes (total)	164	164
	Genes concordant between isolates	—	164/164 (100%)

^†^MLST allelic profile order: *aroC-dnaN-hemD-hisD-purE-sucA-thrA*. ANI, average nucleotide identity; cgMLST, core-genome multilocus sequence typing; MLST, multilocus sequence typing; SISTR, Salmonella *In Silico* Typing Resource; VFDB, Virulence Factor Database. Tools/databases: SISTR (serovar, antigenic formula, cgMLST); mlst (T. Seemann; PubMLST Salmonella scheme, 7-gene MLST); FastANI (pairwise ANI); ABRicate (T. Seemann) with ResFinder database (AMR genes), PlasmidFinder database (plasmid replicons), and VFDB 2022 release (virulence factors).

### Differential antibiotic exposure between the twins

The discordant clinical trajectories of Mo and TB—three acute episodes vs. one—are notable given their shared genetic background and identical household environment. Mo received antibiotics during each of his three recurrences—empirical penicillin (3.10) followed by clindamycin (3.12, during a brief admission at a regional hospital) and then ceftazidime (3.15–3.21) for Episode 1; metronidazole 0.12 g BID for 8 days for Episode 2; and a single 1-day course of clindamycin for Episode 3—cumulatively exposing him to four distinct antibiotic classes. TB, by contrast, received an 8-day course of clindamycin followed by metronidazole 0.12 g BID during his single episode, exposing him to two classes. This differential cumulative antibiotic exposure represents one candidate host-level variable that could be explored in future studies; however, formal causal inference is not possible from a single twin-pair observation, and the observed between-twin difference is equally compatible with chance, unmeasured confounding, or unrelated host factors.

### Exploratory gut microbiota profiling

To explore potential microbiota-level correlates of the differential clinical trajectories, exploratory shotgun metagenomic profiling was performed on 23 longitudinal fecal samples from all five household members, spanning AP and RP timepoints (Figure 1a). LEfSe analysis identified microbial taxa nominally discriminating between acute-phase and reservoir-phase samples at a community level (Figure 1b), with preliminary signals implicating alterations in secondary bile acid metabolic pathways and depletion of butyrate-producing taxa during the acute phase. Species-level composition profiling (Figure 1c) further demonstrated marked dysbiosis in Mo's acute-phase samples, characterized by relative depletion of butyrate-producing commensals including *Faecalibacterium prausnitzii* and *Roseburia hominis*, and concomitant expansion of *Escherichia coli*, a pattern compatible with antibiotic-mediated disruption of colonization resistance. In contrast, TB's samples exhibited comparatively stable community composition across the observation period, paralleling his less recurrent clinical course. These findings are descriptive and hypothesis-generating only; the single-household design and limited sample size preclude population-level inference or causal conclusions.

## Discussion

This case illustrates a clinically important and potentially underappreciated failure mode of standard household outbreak investigation: the systematic inability of conventional direct-plating culture to detect low-level asymptomatic *Salmonella* infection and low-burden carriage. Over a 2-month period involving three recurrent acute NTS episodes in a single child, standard cultures of three co-resident family members (F, M, GM) returned consistently negative results despite F and M experiencing brief, self-limited gastrointestinal symptoms during the outbreak window (GM remained asymptomatic throughout). Solid-phase enrichment applied to the same household-contact specimens also remained negative throughout, although the same enrichment step recovered *Salmonella* from three pediatric acute-phase specimens (Mo 4.12, TB 4.16, and Mo 5.1) that had been negative by conventional culture. Exploratory metagenomic sequencing subsequently detected low levels of *Salmonella*-assigned reads in the household-contact specimens that had been negative by both culture-based methods, a descriptive signal consistent with household-wide low-burden exposure. The sensitivity limitation of conventional culture for low-burden *Salmonella* specimens is well established, with estimates as low as 62%−70% for direct plating in carrier-state scenarios (6). Conventional direct plating is inexpensive, rapid (18–24 h), and broadly available, but its detection threshold (~10^4^–10^5^ CFU/g stool) exceeds the burden typically present in post-convalescent or asymptomatic carriers. Solid-phase enrichment concentrates target organisms from the whole specimen prior to plating, lowering this threshold by approximately two orders of magnitude (7, 8), but adds consumables and turnaround time and has not been standardized across laboratories. Critically, both approaches remain dependent on the recovery of viable organisms from a small fecal aliquot. This limitation is directly relevant to the present case: following acute NTS gastroenteritis, shedding is dynamic and markedly age-dependent, with adults typically excreting only 10^2^–10^3^ salmonellae per gram of stool during convalescence—well within the range that may evade detection by both direct plating and enrichment—while children shed considerably higher loads of 10^6^–10^7^ organisms/g (4). The median duration of post-convalescent fecal shedding in older children and adults is approximately 3–4 weeks, with intermittent excretion rather than continuous shedding (4), such that single-timepoint stool cultures may miss ongoing carriage. In our family, F and M experienced self-limited symptoms days to weeks before the first stool sampling, a timing window in which adult fecal *Salmonella* burdens would be expected to be low and intermittent. The absence of culture positivity in household contacts, even with enrichment, therefore does not exclude intrafamilial transmission; molecular and epidemiological evidence may be the only available means of reconstructing the chain.

The 97% PFGE band-pattern concordance between isolates from Mo and TB was further corroborated by whole-genome sequencing, which demonstrated clonal identity between the two genomes (ANI 99.9966%; identical ST11 MLST and cgMLST profiles; fully concordant resistance-gene, plasmid-replicon, and virulence-factor content). Taken together with the epidemiological context—Mo's recurrences concurrent with F's and M's self-limited gastrointestinal symptoms; absence of an identified external exposure source; metagenomic detection of *Salmonella* DNA in all household-contact specimens—this molecular evidence (97% PFGE similarity, clonal whole-genome concordance between Mo_0412 and TB_0416, and identical antimicrobial susceptibility profiles) supports a common intra-familial source, although microbiological confirmation of carrier status in household contacts remained elusive by both conventional culture and solid-phase enrichment. Notably, ST11 has been consistently reported as the predominant *S*. Enteritidis genotype both in China and globally, accounting for >90% of human isolates in recent large clinical and surveillance cohorts (10, 11). The ST11 background of our cluster therefore reflects the prevailing molecular epidemiology rather than an unusually virulent or atypical clone, arguing against strain-level peculiarity as the explanation for Mo's recurrent disease and refocusing attention on host-level factors (notably cumulative antibiotic exposure) and on the detection-sensitivity limits of conventional culture for low-burden specimens.

The antibiotic exposure asymmetry between Mo and TB invites mechanistic hypothesis generation. Experimental and observational studies have shown that antibiotics can disrupt the commensal microbiota-dependent colonization resistance that normally constrains *Salmonella* establishment and expansion (12, 13). In particular, repeated antibiotic courses have been reported to deplete butyrate-producing commensals—including *Faecalibacterium prausnitzii* and *Roseburia hominis*—which in turn may reduce colonocyte butyrate availability, impair luminal oxygen restriction, and create conditions more permissive to *Salmonella* persistence (14). Whether this framework explains the differential clinical course observed in Mo vs. TB, however, cannot be determined from the present observations: pre-infection baseline microbiota data are lacking, and with only a single twin pair, neither effect size nor direction can be reliably estimated. We therefore present this mechanism strictly as a hypothesis to be tested in prospective cohorts, rather than as an explanation of the present case.

Exploratory metagenomic profiling of 23 longitudinal fecal samples from all five household members provides preliminary descriptive signals regarding phase-associated microbiota dynamics, including alterations in secondary bile acid metabolic pathways. LEfSe analysis identified microbial taxa nominally discriminating between acute-phase (AP) and reservoir-phase (RP) samples. These observations are descriptive and hypothesis-generating only; the single-household design, limited sample size, absence of pre-infection baseline sampling, and lack of a non-exposed control preclude any causal, mechanistic, or population-level inference. In particular, they should not be interpreted as evidence that antibiotic exposure caused Mo's recurrences via loss of colonization resistance, which remains a hypothesis requiring confirmation in larger prospective cohorts with standardized microbiome sampling and appropriate controls (15).

Our experience in this cluster highlights complementary strengths and limitations of the available diagnostic approaches. Conventional direct-plating culture is inexpensive, well standardized, and sufficient for high-burden acute-phase specimens, but its sensitivity declines as bacterial burden decreases—as illustrated by the three pediatric culture-negative / enrichment-positive samples in this cluster (Mo 4.12, TB 4.16, and Mo 5.1). Whole-sample solid-phase enrichment improved yield in these borderline pediatric acute-phase specimens by concentrating target organisms from the entire fecal matrix, but did not detect *Salmonella* in any of the adult-contact specimens, consistent with the intermittent and typically low-level shedding reported in post-convalescent and chronic NTS carriers, in whom fecal *Salmonella* loads can fall below the limit of detection of culture-based assays and fluctuate between samples (4, 16). Nucleic-acid-based approaches such as metagenomic sequencing can detect pathogen DNA below the culture limit, as observed here for F, M, and GM, but do not distinguish viable organisms from residual or transient DNA exposure. From a clinical practice standpoint, this suggests that recurrent pediatric NTS without an identified external source warrants (i) solid-phase enrichment of borderline pediatric specimens to improve acute-case ascertainment and (ii) consideration of molecular methods for household contacts when culture-based testing is uninformative. Evidence-based guidelines for managing asymptomatic or post-convalescent contacts in pediatric household settings remain to be established and represent an important gap for future prospective work.

Although solid-phase enrichment did not directly detect *Salmonella* in household-contact specimens, its recovery of *Salmonella* from Mo 4.12, Mo 5.1, and TB 4.16—three pediatric acute-phase samples otherwise negative by conventional culture—was a prerequisite for the downstream PFGE and whole-genome sequencing analyses on which reconstruction of the household transmission chain ultimately depended. The discordant recurrence pattern between the monozygotic twins generates a testable hypothesis for prospective study, namely whether asymmetric antibiotic exposure and consequent loss of microbiota-mediated colonization resistance modulate susceptibility to recurrent NTS infection; the present single-case observation cannot itself establish such a link. Clinicians investigating recurrent pediatric NTS infections should consider solid-phase enrichment of pediatric specimens to improve acute-case ascertainment and pair it with nucleic-acid-based methods for household-contact screening when conventional cultures are negative but epidemiological context suggests household transmission.

## Data Availability

The raw data supporting the conclusions of this article will be made available by the authors, without undue reservation.
